# Water oxidation by a dye-catalyst diad in natural sunlight: timing and coordination of excitations and reactions across timescales of picoseconds to hours†

**DOI:** 10.1039/d2sc06966k

**Published:** 2023-01-23

**Authors:** Ramzi N. Massad, Thomas P. Cheshire, Chenqi Fan, Frances A. Houle

**Affiliations:** a College of Chemistry, University of California, Berkeley Berkeley CA 94720 USA; b Chemical Sciences Division, Lawrence Berkeley National Laboratory Berkeley CA 94720 USA fahoule@lbl.gov

## Abstract

The mechanisms of how dyes and catalysts for solar-driven transformations such as water oxidation to form O_2_ work have been intensively investigated, however little is known about how their independent photophysical and chemical processes work together. The level of coordination between the dye and the catalyst in time determines the overall water oxidation system's efficiency. In this computational stochastic kinetics study, we have examined coordination and timing for a Ru-based dye-catalyst diad, [P2Ru(4-mebpy-4′-bimpy)Ru(tpy)(OH_2_)]^4+^, where P2 is 4,4′-bisphosphonato-2,2′-bipyridine, 4-mebpy-4′-bimpy is 4-(methylbipyridin-4′-yl)-*N*-benzimid-*N*′-pyridine, a bridging ligand, and tpy is (2,2′:6′,2′′-terpyridine), taking advantage of the extensive data available for both dye and catalyst, and direct studies of the diads bound to a semiconductor surface. The simulation results for both ensembles of diads and single diads show that progress through the generally accepted water oxidation catalytic cycle is not controlled by the relatively low flux of solar irradiation or by charge or excitation losses, rather is gated by buildup of intermediates whose chemical reactions are not accelerated by photoexcitations. The stochastics of these thermal reactions govern the level of coordination between the dye and the catalyst. This suggests that catalytic efficiency can be improved in these multiphoton catalytic cycles by providing a means for photostimulation of all intermediates so that the catalytic rate is governed by charge injection under solar illumination alone.

## Abstract 2

Excitations and reactions in a dye−catalyst dyad for solar-driven water oxidation have timing mismatches caused by the stochastics of some catalytic steps. Rather than being perfectly meshed, these processes are like gears with missing teeth.

## Graphical abstract

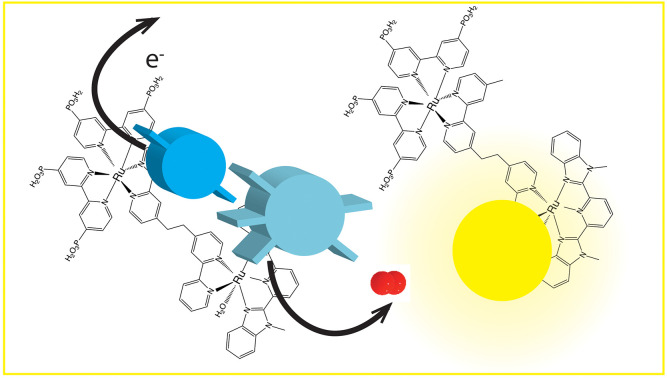

## 1 Introduction

Photoelectrodes for dye-sensitized photoelectrosynthesis cells (DSPECs) are highly flexible architectures for conversion of solar energy to chemical energy, offering the potential to precisely tailor both sensitivity to light and catalytic efficiency and selectivity at the same time.^1–3^ They involve two primary molecular components: a dye that absorbs light and becomes oxidized or reduced, and a catalyst that is activated by transferring an electron to the dye, thus initiating a step in the catalytic cycle. The dye is anchored to a semiconductor substrate to which it injects charge, leading to its change in oxidation state. Electron transfer to the catalyst can be substrate-mediated if the catalyst and dye are individually attached to the semiconductor. If the catalyst is covalently linked to the dye to form a diad, however, the electron transfer process between catalyst and dye is direct, which is the focus of this manuscript.

The value of the dye-catalyst diad construct lies in its potential to operate in a self-contained way with sunlight as the sole source of energy, and therefore its efficiency must be as high as possible. Three independent factors control the efficiency of a diad: the *quantum yield for charge injection* by the dye, the *rate of each step in the catalytic cycle*, and the *coordination between sporadic excitations by sunlight and the cadence of the catalytic cycle, which is inherently stochastic*. In this work, we specifically examine these factors for linked dye-catalyst diads that can perform sunlight-driven water oxidation: the 4-photon process of converting 2 water molecules to O_2_, protons and electrons. Highly efficient dyes will have near unity quantum yield for charge injection, so each solar photon absorbed will result in dye oxidation, setting the catalyst up to be activated. The rates of reaction of the activated catalysts as they advance through the cycle depend on the rate coefficients and on the instantaneous concentrations of the various catalyst intermediate states, which can vary in time. Although these first two factors have been intensively investigated separately, the details of how the dye and catalyst's separate processes are coordinated when they work as a pair, and how this coordination can affect catalytic or photo efficiency (or both) are less well understood. This is because of the considerable experimental challenges involved in directly observing a multistep catalytic process, particularly when it is controlled by diffuse illumination (sunlight), and the theoretical challenges posed by having to span broad timescales.

Ideally, the diad would function like two gears in a machine, with the dye driving progress through the catalytic stages and ensuring coordination between excitation and reaction. How well this works in practice depends on two characteristics. First, charge transfer must be very efficient, with few avenues for losses. Although the quantum yield for charge injection from the most efficient dyes is high, back electron transfer (BET) from the dye and possibly also the catalyst (which is more distant) to the semiconductor is a very important process that can interrupt a step in the catalytic cycle by returning the newly prepared intermediate back to an unreactive state.^4–6^ The second characteristic involves the timing of excitations and charge injection relative to the bond-making and -breaking steps involved in each stage of the catalysis. A key aspect of understanding timing is recognizing that when reactions are triggered by low intensity light such as solar irradiation, they may only occur sporadically^7,8^ leading to a single reaction step, such as dissociation, or a sequence of steps, then a pause until the next photon is absorbed. If each reaction step in the catalytic redox cycle is completely photo-driven, progress through it will be gated by these sporadic photoexcitation events. More commonly, photoexcitation initiates a series of non-photodriven steps (thermal and charge transfer events) associated with a catalytic stage. The degree of coordination between the dye and the catalyst therefore depends on how much of the catalysis is directly driven by light, as well as on the timings of the non-photodriven steps which are stochastic on a molecular level.

**Fig. 1:**
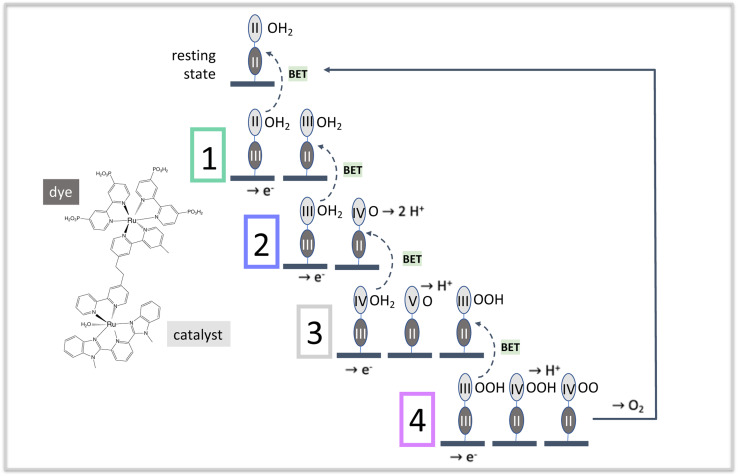
The four stages of water oxidation catalysis driven by photoexcitation of dyes in the Ru^II^ state. A photoexcitation step begins each cycle, oxidizing the dye in the diad product of the preceding step. The color scheme, used throughout this paper, is as follows: dye: dark gray; catalyst: medium gray; resting state of the diad: black; stage 1: gray-green; stage 2: dark lavender; stage 3: light gray; stage 4: rose. The dashed arrows connecting a stage to its preceding one denote back electron transfer processes that reduce the oxidized dye or the oxidized catalyst.

In the present study, we characterize coordination in two ways. In the first we simulate the water oxidation reaction when a large ensemble of diads is involved, for example on a nanoparticulate semiconductor support. In the second, using the same kinetic mechanism, we calculate the time evolution of a single diad in sunlight, which reveals details of the timings, controlled by the stochasticity of all the events involved in the catalytic cycle. All redox states of the dye and catalyst are tracked throughout the full catalytic cycle, using stochastic kinetics simulation methods^9,10^ and mechanistic data taken from the literature. This approach requires an accurate kinetic scheme, and therefore we focus on a well-studied model diad system couples a Ru-based dye with a Ru-based catalyst (Fig. 1), where detailed mechanistic data are available for both the dye photophysics^11–15^ and the catalytic redox cycle.^5,16–21^ This catalyst is not efficient for water oxidation especially at low pH,^21^ indeed superior catalysts are known,^3,22^ however it and its close analogs are the only ones for which there is sufficient information available to construct a physically and chemically detailed, predictive model.

Fig. 1 illustrates the catalytic cycle for this type of diad in acidic solution, and includes the structure of one dye-catalyst combination,^23^ [P2Ru(4-mebpy-4′-bimpy)Ru(tpy)(OH_2_)]^4+^, where P2 is 4,4′-bisphosphonato-2,2′-bipyridine, 4-mebpy-4′-bimpy is 4-(methylbipyridin-4′-yl)-*N*-benzimid-*N*′-pyridine, a bridging ligand, and tpy is (2,2′:6′,2′′-terpyridine).^24^ The mnemonic used to describe the states of the diad in all tables and figures in this work is Ru^dye oxidation state^–Ru^catalyst oxidation state^(oxygen state). For example, the resting state of the catalyst is Ru^II^–Ru^II^(OH_2_).

Starting from the diad's resting state, Ru^II^–Ru^II^(OH_2_), one photoexcitation – charge injection – intra-diad charge transfer step at the beginning of each of the 4 stages excites the dye from the Ru^II^ state followed by charge injection and formation of the Ru^III^ state. This state converts back to the Ru^II^ state after charge transfer from the catalyst, increasing the catalyst's oxidation state. Most of the work of dissociating two water molecules and forming an O_2_ bond takes place when the catalyst is in the Ru^IV^ and possibly the Ru^V^ state,^5,18,19,25^ so the dye's purpose is to populate those states. The coordination of the dye-catalyst diad throughout all of these elementary processes depends on how promptly this purpose is fulfilled at each stage of the catalysis, which in turn strongly influences efficiency. The simulations reported here provide important new insights to how this class of diads functions: their catalytic efficiency is not limited by BET or similar losses, contrary to what has been proposed in the literature.^6,26–31^ We show that efficiency is limited by the lack of a mechanism to coordinate fully photoexcitation with catalysis when the two processes take place at separate molecular centers. This finding has general implications for the design of effective diad constructs as well as substrate-mediated redox reactions.

## 2 Model construction

In a DSPEC, the diads are anchored to semiconductor nanoparticles that are sintered into a porous film on a transparent conducting electrode illuminated by the sun.^23^ The dye is attached to the nanoparticles by phosphonate groups, and the catalyst is attached to the dye using a linker. Aqueous electrolyte infiltrates the photoanode's pores. Electrons injected into the nanoparticles percolate through the porous film of the photoelectrode, from where they can be collected and used to drive reduction chemistry at a cathode in a full system. Although the details of the illuminated porous photoanode and the electrolyte can be included in full,^32,33^ for the purposes of this work the simulation focuses only on the local molecular-level details taking place at the nanoparticle surfaces, ignoring the pore environment *per se*. This is possible because water is in vast excess over the diad concentrations so its availability is not transport-limited. We assume that the local pH is maintained constant within the pore environment, and that the photoanode is uniformly illuminated throughout its thickness, which is not unreasonable when the porous layer is a few μm thick.^24^ These assumptions allow three useful model simplifications: the system is zero-dimensional, the semiconducting nanoparticles on which the diads are adsorbed are implicit, and the kinetic dependences of the chemistry on both the solar flux as a function of wavelength and the electrolyte are treated as pseudo-first order. The most complete set of kinetic data for the catalyst has been measured at pH 1, therefore the simulation results are most relevant to acidic conditions. Because the sequence of redox steps in the catalytic mechanism is qualitatively the same under less acidic conditions,^24^ extension of the model to them is straightforward if the rate coefficients are available.

### 2.1. System characteristics

The zero-dimensional compartment used for the calculations contains 2 × 10^−3^ moles cm^−3^ of dye-catalyst diads, adsorbed in a 1 nm thick monolayer in the high surface area nanoparticulate film. This corresponds to the diad concentration reported in experimental studies in the range of 2 × 10^−8^ moles cm^−2^, where the area is the geometric area of the nanoparticulate sample.^5^ The coverage in the 1 nm thick layer is 1.2 × 10^14^ diads per cm^2^, or about 1 diad in 0.8 nm^2^. In addition to the diad ensemble simulations, we have performed a set of simulations for a single diad adsorbed on 1 nm^2^ using the same reaction mechanism. Since the semiconductor is implicit and conduction paths are not provided to remove the accumulating electrons after they are injected, the electron population generated by the dyes is assumed to be constant at 4 × 10^17^ cm^−3^.^6^ The details of the kinetic steps and the simulation methodology are as follows.

### 2.2. Dye photophysics

The kinetics of photophysical processes including charge injection that have been extracted using simulations of transient absorption data to represent the dye, and have been reported in full elsewhere.^11–13^ Briefly, the experimental ultrafast transient dye photophysics for a family of Ru polypyridyl dyes in methanol or acetonitrile solutions have been reproduced using detailed kinetics models, and the kinetics have been used to predict excitations under steady state solar irradiation. Using the molecular schemes, the photophysics for these dyes attached to Zr and Ti oxide surfaces have been determined, including the charge injection rate coefficients. The ground state is excited into the singlet manifold, consisting of three states, Y, B and X, where Y has the highest energy and X has the lowest, and directly to the triplet state. The precise identity of these states has not yet been determined. Relaxations from the Y to B and B to X states precedes intersystem crossing to the triplet manifold from state X. Because the effect of changing the solvent from methanol to water on the primary electronic transitions in solution appear to be small,^34^ the photophysical kinetics are assumed to apply to the aqueous environment. Illumination by continuous sunlight is isotropic, and the incident light intensity is that of the solar spectrum integrated from 375–750 nm, which is the range absorbed by the dye complexes. The photophysical steps for the specific dye used in the diads, RuP2, are presented in Table 1 and in more detail in Table S1 (ESI†). The excitation rate coefficients for each transition are pseudo-first-order: the transition rate coefficients (in the range of 10^13^ s^−1^) are multiplied by the fractional photon population in the relevant wavelength range for that transition in order to avoid the computational cost of including the photon flux explicitly. They are corrected for optical scatter in the nanoparticulate film.

**Table 1:** Photoexcitation steps and rate coefficients for RuP2 dye under continuous solar illumination

Process	Excitation step	Rate coefficient^a^
Dye excitation	Ru^II^ → singlet Y	4.28 s^−1^
	Ru^II^ → singlet B	13.2 s^−1^
	Ru^II^ → singlet X	22.8 s^−1^
	Ru^II^ → triplet	18.3 s^−1^
Ground state bleach	*Via* singlet Y	4.28 s^−1^
	*Via* singlet B	13.2 s^−1^
	*Via* singlet X	22.8 s^−1^
	*Via* triplet	18.3 s^−1^
Simulated emission	Singlet Y → Ru^II^	4.28 s^−1^
	Singlet B → Ru^II^	13.2 s^−1^
	Singlet X → Ru^II^	22.8 s^−1^
	Triplet → Ru^II^	18.3 s^−1^
Internal conversion	Singlet Y → singlet B	2.4 × 10^13^ s^−1^
	Singlet B → singlet X	2.4 × 10^13^ s^−1^
Intersystem crossing	Singlet X → triplet	4 × 10^13^ s^−1^
Excited state absorptions	Singlet Y	117 s^−1^
	Singlet B	117 s^−1^
	Singlet X	117 s^−1^
	Triplet	117 s^−1^
Incoherent emission	Triplet → Ru^II^	9.6 × 10^4^ s^−1^
Nonradiative relaxation	Triplet → Ru^II^	2.6 × 10^6^ s^−1^
Charge injection to substrate	Singlet Y → Ru^III^ + electron	1 × 10^12^ s^−1^
	Singlet B → Ru^III^ + electron	1 × 10^12^ s^−1^
	Singlet X → Ru^III^ + electron	1 × 10^12^ s^−1^
	Triplet → Ru^III^ + electron	1 × 10^12^ s^−1^
Back-electron transfer from substrate	Ru^III^ → Ru^II^	8 × 10^−6^ s^−1^ (ref. 6)

a Coefficients are pseudo-first order in light intensity integrated over the wavelength range for the excitation.

The back electron transfer (BET) rate coefficient has been of considerable discussion in the literature because of the potential impact of electron losses on catalytic efficiency, the complex kinetics involved, and the influence of potential and measurement conditions on value reported.^4,6,26,28,35^ Using diverse experimental techniques, second order rate coefficients for electron-oxidized dye recombination of 300 cm^3^ per mole per s ^4^ and 12 cm^3^ per mole per s ^6^ have been reported. In the present study, we focus on the smaller value because it is more relevant to low light fluxes, however for comparison we have also performed full simulations under additional BET scenarios, as described in the Results section. The second order BET rate coefficient is converted to a rate coefficient that is pseudo-first order in electrons, 8 × 10^−6^ s^−1^, using the typical experimentally determined electron density of 4 × 10^17^ cm^−3^ = 6.6 × 10^−7^ moles cm^−3^.^6^

### 2.3. Water oxidation catalysis

The mechanism for water oxidation catalysis by this class of complexes has been extensively studied using both photoexcitation (through the first two steps of the cycle) and oxidation of the catalyst's intermediate states by Ce^4+^ (the complete cycle). Most of these studies have been in homogeneous solutions, with some investigations of heterogenized catalysts.^5,16–18,20^ The catalyst reaction steps used in the simulations are shown in Table 2. The rate coefficients for each reaction step are taken from studies performed in 0.1 M aqueous nitric acid, except for the dye-catalyst electron transfer step which was measured in aqueous 0.1 M HClO_4_. It is worth noting that the elementary steps for how stages 3 and 4 work together, and whether the dissociation of the peroxy species has in fact such a slow rate coefficient, are complex^5,36^ and have not been fully delineated including the relevant detailed rate coefficients. We chose here to use rate coefficients determined for photodriven diads wherever available. The results of the simulations reported here may be affected in detail by improved descriptions of the catalysis, especially in predicting which Ru^II^–Ru^IV^ intermediates are persistent and observable spectroscopically, but the overall findings on how the functions of the dye and the catalyst are coordinated are unlikely to change.

**Table 2:** Catalyst reactions using 4 electrons to form 4 protons and O_2_ following charge injection from the dye

Catalytic cycle		Reaction	Rate coefficient
Stage	Step		
1	a (oxidation)	Ru^II^–OH_2_ → Ru^III^–OH_2_ + electron	6.9 × 10^9^ s^−1^ (ref. 15)
	b (BET)	Ru^III^–OH_2_ + electron → Ru^II^–OH_2_	8 × 10^−6^ s^−1^ (ref. 6)
2^a^		Ru^III^–OH_2_ → Ru^IV^=O + 2H^+^ + electron	0.036 s^−1^ (ref. 5)
3	a (oxidation)	Ru^IV^ = O → Ru^V^=O + electron	6.9 × 10^9^ s^−1^ (ref. 15)
	b	Ru^V^ = O → Ru^III^–OOH + H^+^	9.6 × 10^−3^ s^−1^ (ref. 17)
	c (BET)	Ru^V^ = O → Ru^IV^=O	8 × 10^−6^ s^−^ (ref. 6)
4	a (oxidation)	Ru^III^–OOH → Ru^IV^–OOH + electron → Ru^IV^OO + H^+^	6.9 × 10^9^ s^−1^ (ref. 15)
	b	Ru^IV^–OO → Ru^II^–OH_2_ + O_2_	7.5 × 10^−4^ s^−1^ (ref. 17)
	c (BET)	Ru^IV^–OOH → Ru^III^–OOH	8 × 10^−6^ s^−1^ (ref. 6)

a Step 2 is only reported as a composite step for the Ru–Ru diad.

Dye-catalyst charge transfer, which oxidizes the Ru in the catalyst to drive the water splitting process, is represented explicitly in steps 1a, 3a and 4a. It was measured using transient absorption spectroscopy for a diad that uses the same catalyst with a related dye and a more complex linker.^15^ The oxidation and proton loss processes in stage 2 have been investigated using photoexcitation not been kinetically resolved, so are combined as a single step.^5^

The rate coefficients for BET involving the catalyst, steps 1b, 3c and 4c,^28^ have not been reported, so the rate coefficient for the dye-substrate back electron transfer process has been used as an upper limit (Table 1).

The reaction rate coefficient for step 3b has been shown by electrochemistry to be very sensitive to the presence of basic species in the electrolyte,^16^ so this value is a lower limit. The studies in which Ce^IV^ is used as an oxidant are less easily interpreted past the 2nd stage of the catalytic cycle, presumably due to complicating factors during the reactions that are not present under (photo)electrochemical conditions.^17–20^ Nonetheless, all studies agree that at a pH of 1 in HNO_3_ the final step, loss of O_2_, has the smallest rate coefficient, with values of 1.2 × 10^−4^ s^−1^,^20^ 4.9 × 10^−4^ s^−1^,^18^ and 7.5 × 10^−4^ s^−1^.^17^ The last of these is used in this work in order to be internally consistent with step 3b, which came from the same study.

### 2.4. Simulation methodology

Stochastic kinetics simulations have been performed using the open-access package Kinetiscope.^37^ The stochastic method^9,38^ is well-suited to multiscale simulations that link molecular-level events to experimental observables.^39^ It is a rigorous solution to the master equation, and because it uses simple arithmetic rather than coupled differential equation integration, it is capable of simulating systems that combine a very broad range of timescales in a single simulation, spanning ps to nearly an hour for the ensemble diad system and ps to 24 hour for the single diad. The flexibility with which mechanisms can be set up supports an inductive approach to model building.^40^ This approach is particularly useful for constructing complex mechanisms from sub-mechanisms that span inherently disparate timescales such as the one presented here, as well as identifying where gaps in knowledge exist, and identifying where new experiments and theoretical calculations can fill them.

Simulations are propagated by random selection of probability-weighted steps in the reaction mechanism, and the time steps are calculated from the instantaneous rates of the steps. Accordingly, if accurate rate coefficients for each step are used in the simulations, an absolute time base can be generated and the simulations can be analyzed to generate data that can be directly compared to experimental results and used to gain insights to stochastic and sporadic processes. A significant advantage to the stochastic method is that marker species can be embedded throughout the reaction scheme, enabling a deeper analysis of the reaction than is possible with computing concentrations of chemical species alone. The occurrences of specific steps can be counted using these markers, and the rates of those steps as a function of time can be calculated by taking the derivative of the accumulated marker quantities as a function of time. The markers used in the present work track all the steps in Tables 1 and 2, and are presented together with the full scheme for the diad kinetics in ESI Section 1 and Table S1.†

In the present study, simulations of diad ensembles were performed up to a total time period of 2400 s, consisting of 1200 s of solar illumination followed by 1200 s in the dark. Four sets of calculations examine the influence of BET as described below. Simulations of a single diad were performed for 8 hours of solar illumination followed by 16 hours of darkness. 20 single diad simulations with different random number sequences allow a set of 397 full catalytic cycles to be captured, enabling statistical analysis of the time lapse between specific events at the molecular level. The purpose of using a light–dark sequence is to be able to follow the chemistry both while the catalytic cycle is being driven and when it is relaxing in order to learn how all the redox states evolve in time, which is relevant to operation under intermittent insolation. Real systems can have significant losses, leading to low turnover numbers.^41^ Because the relevant mechanistic data are not available, losses are not included here.

## 3 Results

### 3.1. Overall efficiency of the diad: ensemble simulations

**Fig. 2:**
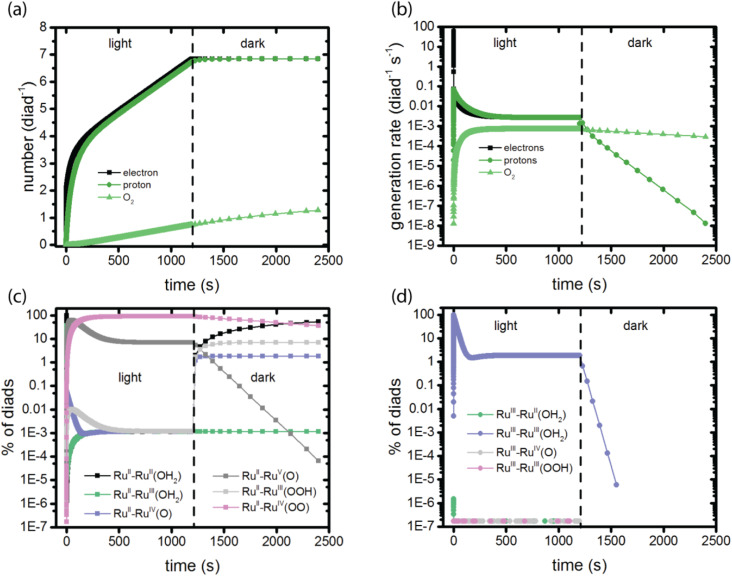
Observables predicted by the simulations in a 1200 s light – 1200 s dark sequence. (a) Total electrons, protons and O_2_ generated per diad; (b) rate of electron, proton and O_2_ generation per diad per second; (c) diads present with dye in the Ru^II^ state; (d) diads present with the dye in the Ru^III^ state. The populations in panels (c) and (d) together add up to 100%. The colors correspond to the species present in each catalytic stage, Fig. 1.

The calculations provide data for the total net rate of formation of protons, electrons and O_2_ generated per dye during the catalytic cycle as a function of time, as shown in Fig. 2a and b. Net rates are the derivatives of the time-histories of each species, which are the primary simulation results, and depend on the rate coefficients as well as the instantaneous reactant populations for all steps leading to their formation and consumption. The initial rate of generation of electrons and protons is very high, dropping rapidly to lower values within a few seconds. The electron generation rate is initially faster than the proton generation rate. This is because stage 1 in the catalysis (Fig. 1), which does not generate a proton, dominates the kinetics at early times. Proton and electron generation only become balanced when steady state is reached between 500 and 1000 s. The total numbers of electrons and protons produced per diad in 1200 s are 6.8 and 6.7, respectively. Based on these numbers, the total O_2_ per diad would be expected to be about 1.7 (1/4 of the total), however the calculations predict that 0.74 would be present. Fig. 2c explains this result: the buildup of Ru^II^–Ru^IV^(OO) accounts for about 0.91 O_2_ per diad in the system, totaling 1.65 O_2_ in the dioxygen and peroxy states overall. The remaining 0.05 oxygens per diad are sequestered in the other diad states. Proton and O_2_ generation continue in the dark because of the slow spontaneous reactions of the intermediates present after illumination stops. The steady state O_2_ production rate corresponds to a photodriven turnover frequency (TOF) for the diad of 6.8 × 10^−4^ s^−1^. This value is in the same range of the values for the O_2_ production frequency measured for this one and related homogeneous catalysts,^20,42,43^ 1.4 × 10^−4^ – 1.4 × 10^−3^ during electrochemical water oxidation, indicating that the catalyst portion of the diad controls the TOF, not the dye.

### 3.2. Catalytic intermediates: ensemble simulations

The molecular features of the time-dependence of catalytic product formation are shown in Fig. 2c (diads with ground state dyes) and 2d (diads with oxidized dyes). The populations span many orders of magnitude under illumination and vary with time. It can be seen from the plots that even though the production of electrons, protons and O_2_ appear to have reached steady state by 500 s, the distribution of intermediates does not reach steady state until later, closer to 1000 s (Fig. S1, ESI†). At steady state 90.99% of the diads are in the Ru^II^–Ru^IV^(OO) form, generated during stage 4 of the catalysis (Fig. 1), with Ru^II^–Ru^V^(O) and Ru^III^–Ru^III^ (OH_2_) being the other dominant intermediates at 7.11% and 1.89%, respectively. The remaining 0.01% of the diads is distributed across 7 additional oxidation states. When illumination stops, the diad population slowly decays back to a mixture of mainly Ru^II^–Ru^II^(OH_2_) and Ru^II^–Ru^III^(OOH) as O_2_ is released. There are literature reports that Ru^IV^ forms of the catalyst are quite persistent even when the catalysis reaction has been stopped,^25^ but not the Ru^III^(OOH) species, which has been pointed to as being unstable, decomposing to Ru^II^(OOH).^17^ A study of homogeneous catalysts has found that Ru^IV^(OO) requires the presence of an oxidant to release O_2_.^25^ Loss processes for the Ru^II^–Ru^III^(OOH) diad would have to be included to agree with experimental observations. The chemical identity of the dominant persistent Ru^IV^ species has been reported to be Ru^IV^(OO) or Ru^IV^(O) or both when generated using Ce^IV^ oxidants, electrochemistry and photooxidation.^5,17,25,43^ A comprehensive theory and experimental study has reported spectroscopic signatures of different Ru^IV^ forms at different points in the catalytic cycle.^25^

On comparing the time variation of the intermediates in Fig. 2 as the reaction progresses to steady state, it is clear there is a progression from of the dominant intermediate starting from Ru^II^–Ru^II^(OH_2_), to stage 2, Ru^II^–Ru^IV^(O), to stage 3, Ru^II^–Ru^V^(O) and Ru^II^–Ru^III^(OOH), each of which go through a peak then decline in concentration. Ru^II^–Ru^IV^(OO) (stage 4) and small amounts of Ru^II^–Ru^III^ (stage 1) build up to their steady state values. The persistent concentration of the Ru^V^(O) species is predicted by the kinetic mechanism and rate coefficients reported in the literature, however this has not been universally found in experimental measurements on diads. Measurements on specific related catalysts have reported both its presence^17,18,25,44^ and its absence.^19^

To our knowledge only one publication reports direct observations of the speciation of these diads under illumination as a function of time.^5^ In that work, the Ru^II^–Ru^III^(OH_2_) diad was used as the starting state, and spectroscopic measurements were used to identify species present under irradiation in yellow light at 100 mW cm^−2^ for 600 s, which mainly excites the dye directly into its triplet state.^13^ The species observed by resonance Raman spectroscopy were assigned to Ru^II^–Ru^II^(OH_2_), Ru^III^–Ru^III^(OH_2_) and Ru^II^–Ru^IV^(O), and with additional rather intense features in the spectra potentially arising from a peroxide species. This suggests that the diad only executed one catalytic cycle under those experimental conditions, because the Ru^II^–Ru^II^(OH_2_) resting state should have been rapidly oxidized to start a new cycle. Indeed, the product distribution is closer to that observed in the dark (Fig. 2). It would be useful to have additional experimental data to compare to the simulation results: such a comparison will help refine the model framework and its mechanistic steps.

### 3.3. Dye excitations and charge injection: ensemble simulations

**Fig. 3:**
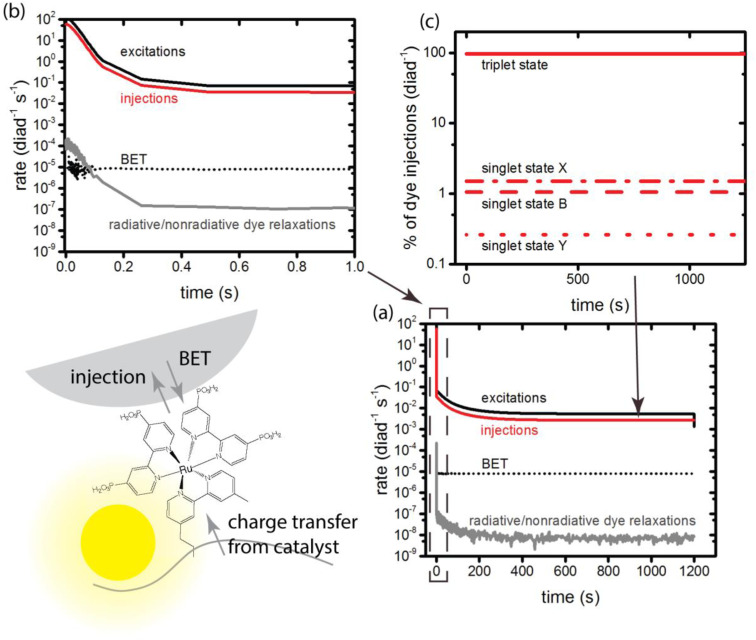
Primary photophysical and loss processes of the dye component of the diads, totals for all states under illumination. (a) Rate of excitation, charge injection, back electron transfer, and radiative/nonradiative losses from the dye's excited singlet and triplet states; (b) detail of (a), showing changes during the first second of illumination; (c) details of total charge injections in (a), showing the identity of the injecting state.

At the very earliest times, up to about 1 ms, the dye excitation and electron injection rates are nearly constant, with values of about 117 excitations per diad per s and about 58 electron injections per diad per s as shown in Fig. 3a and b. Fig. 3c shows the breakdown of the injecting states: charge injection from the triplet state, whether directly populated by absorption or by intersystem crossing, is dominant. Assuming all injections lead to catalytic steps and that the photophysics controls the diad's progress through the catalytic cycle, the highest TOF that could be expected for the 4-photon process in sunlight is about 15 s^−1^. At steady state, the BET rate is essentially constant at 8 × 10^−6^ per diad per s, compared to the total injection rate of about 2.7 × 10^−3^ per diad per s.

**Fig. 4:**
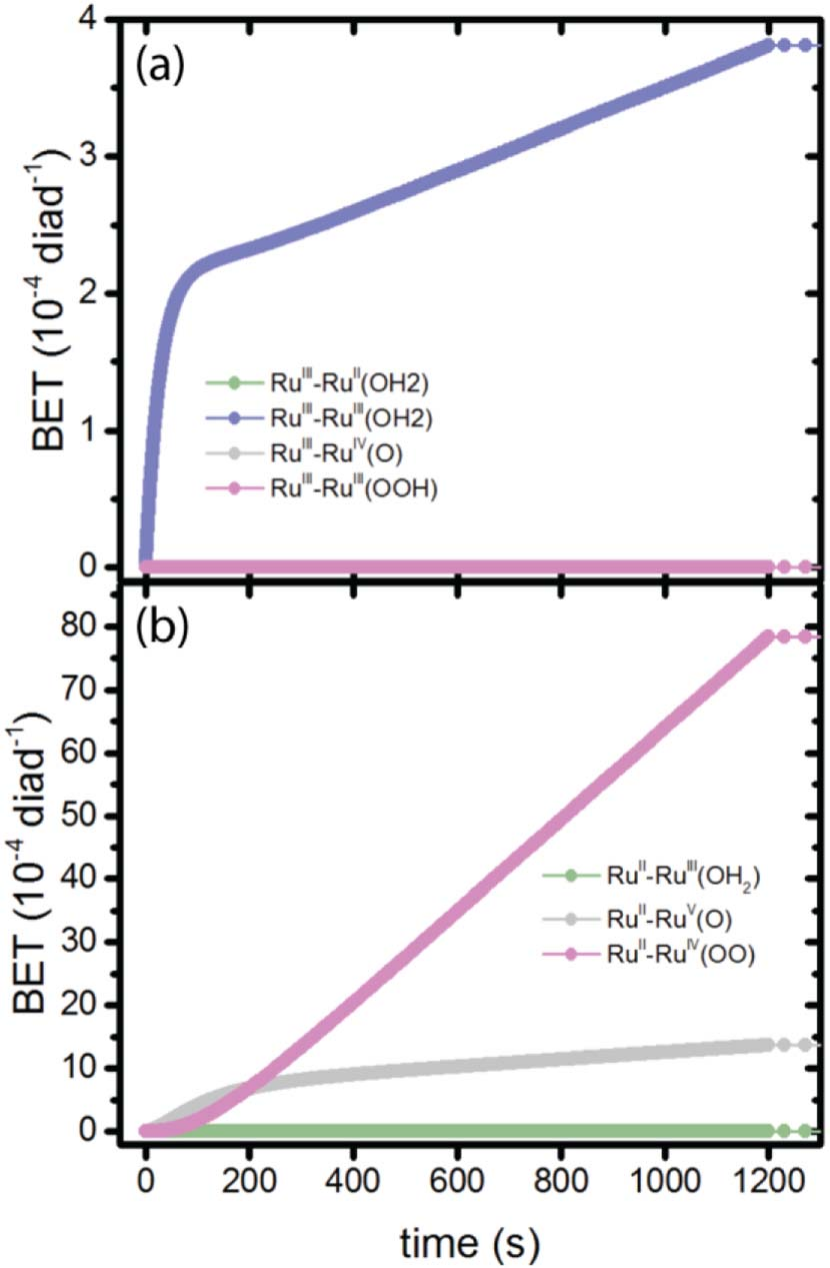
Total number of BET events for all diads as a function of time, shown for each diad species. (a) BET to the dye, initially in the Ru^III^ state; (b) BET to the catalyst, initially in the Ru^III^, Ru^V^ and Ru^IV^ states.

There is disagreement in the literature about the BET rate coefficient, not only concerning its magnitude, but also whether or not it should be second order in molecules and electrons.^4,6^ Here we assume it is second order in electrons and diads, and examine 4 scenarios for BET in order to understand how the magnitude of the BET process may influence catalysis. Scenario 1 is the base mechanism in Tables 1 and 2. Scenario 2 uses a faster rate coefficient (pseudo first order value of 2 × 10^−4^ s^−1^ from pulsed laser measurements)^4^ for both catalyst and dye back electron transfer, Scenario 3 uses the same faster rate coefficient for dye back electron transfer only, assuming transfer to the catalyst is very slow,^5,26^ and Scenario 4 assumes no back electron transfer occurs at any time. The total number of BET events occurring during illumination as a function of time for each oxidized species under scenario 1 is plotted in Fig. 4, with Fig. 4a showing BET to the catalyst and Fig. 4b to the dye. It is evident that BET involves a subset of states, those whose populations have been shown to build up in Fig. 2c and d, and that BET to the catalyst is dominant despite conclusions in the literature that it is not significant relative to BET to the dye. This may be a consequence of the rate coefficient assumed in this work, for lack of specific measurements for catalyst BET relative to dye BET. The simulation results have been analyzed to determine the total numbers of BET events per diad, and electrons, protons and O_2_ generated per diad in 1200 s under these 4 scenarios as shown in Table 3. The amount of oxygen generated per diad is unaffected by BET. Scenarios 1, 3 and 4 have the same electron and proton yields per diad, but the values for scenario 2 are larger because BET causes a small fraction of the diads to cycle multiple times through the same catalytic stage. Overall, the percent of diads that undergo BET is very small, indicating that BET is not a significant cause of inefficiency in this class of diads, contrary to what has been proposed.

**Table 3:** Simulation results, comparing total numbers for different BET scenarios in 1200 s

Scenario	*k*_BET_ a	Total BET per diad^b^	Total e^−^ per diad	Total H^+^ per diad	Total O_2_ per diad^c^	% Lost to BET^d^
(1) Both catalyst and dye	8 × 10^−6^ s^−1^ (ref. 6)	0.0096	6.84	6.73	0.74	0.14
(2) Both catalyst and dye	2 × 10^−4^ s^−1^ (ref. 4)	0.24	7.07	6.92	0.74	3.39
(3) Dye only	2 × 10^−4^ s^−1^ (ref. 4)	0.0095	6.84	6.72	0.74	0.14
(4) No BET	0	0	6.83	6.72	0.74	0

a Pseudo first order values.

b Diad amount = 2 × 10^−10^ moles.

c At 1200 s, 0.96 O_2_ per diad is in the form of Ru^II^–Ru^IV^(OO), which decomposes slowly.

d Calculated from the fraction of electrons consumed by BET relative to the total injected electrons.

### 3.4. Coordination and timing, ensemble simulations

**Fig. 5:**
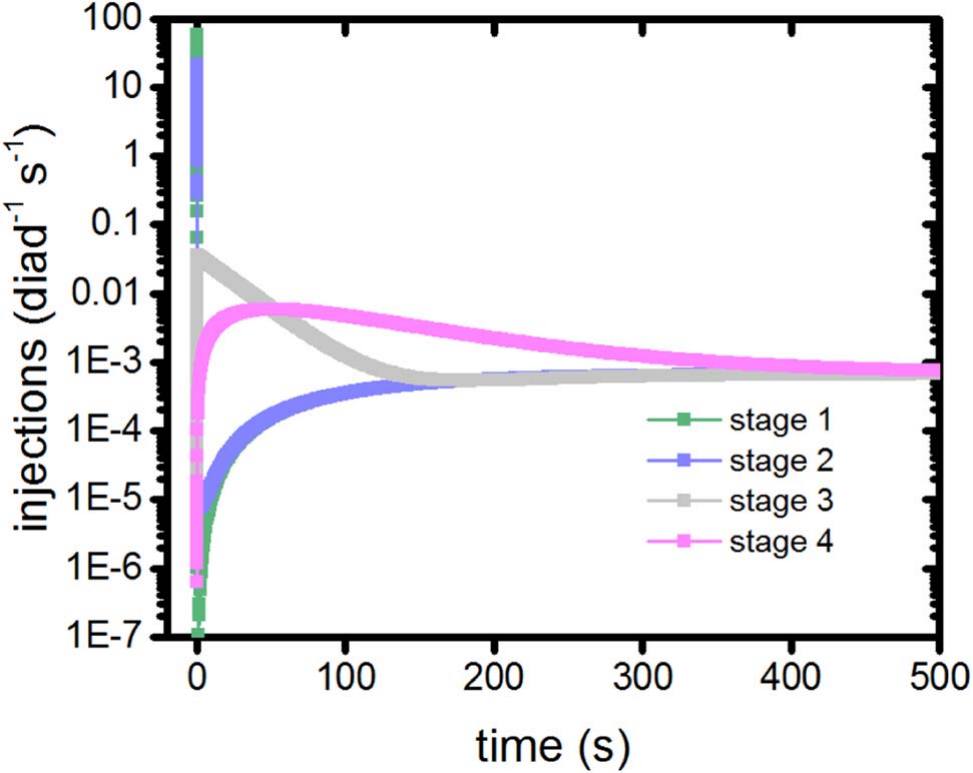
Timings of the catalytic stages as measured by the electron injection step for each diad during that stage.

In this model, dye excitations and charge injection are only tracked when they are associated with a change in redox state of the catalyst (Table S1†). As such, they provide a clock to measure the coordination and timing of each of the catalytic stages. The electron injection steps of a stage from the singlet and triplet states are recorded using markers, allowing the injection rate per diad as a function of time for each stage to be calculated as shown in Fig. 5.

**Fig. 6:**
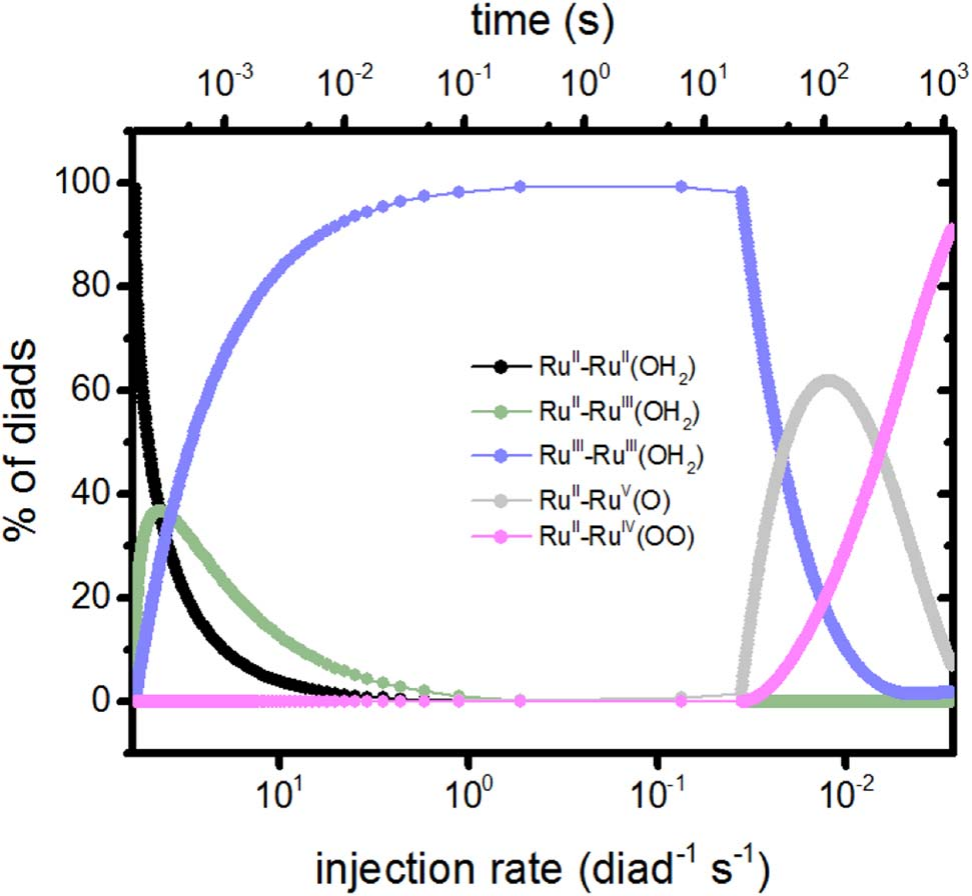
Percents of the diad populations present as a function of time and as a function of the electron injection rate, which decreases rapidly from its initial value (Fig. 2). The non-photoactive intermediate species build up then decline sequentially as the catalysis evolves toward steady state.

The injection rates are a measure of how frequently the catalysis stages are initiated, and are a function of the instantaneous diad populations and the rate coefficients. At the earliest times, the fastest injection rates are in stages 1 and 2, followed by a very steep drop when the Ru^II^–Ru^II^(OH_2_) and Ru^II^–Ru^III^(OH_2_) populations are depleted (Fig. 2c). The diad populations governing the injection rates for stages 3 and 4 evolve more slowly. The injection rates for all 4 stages become similar (7 × 10^−4^ per diad per s) at about 500 s. This marks a steady state. Another view of the timings of catalytic stages as the system evolves toward steady state is shown in Fig. 6, which presents the percentages of the most abundant diads as a function of both the total electron injection rate and time. The total injection rate starts at about 58 per diad per s, declining to 1 per diad per s within a few hundred ms. This drop is accompanied by the transformation of the diad population to be almost entirely Ru^III^–Ru^III^(OH_2_), which is not photoactive. It is reactive, however, and its population eventually declines. By the time that bottleneck is gone, at around 100 s, the injection rate has dropped to 0.006 per diad per s due to the buildup of two additional non-photoactive species, Ru^II^–Ru^V^(O) and Ru^II^–Ru^IV^(OO). Thus, at steady state, the accumulation of >99.9% of the diads into states that can only react thermally completely controls progression of the diad through the stages of the catalytic cycle, and the efficiency of the chemistry. The dye no longer plays its initially central role.

It is noteworthy that although the injection rates appear to reach steady state in about 500 s (Fig. 5), the distribution of diad intermediates takes longer to stabilize, to about 1000 s (Fig. 6). This places significant constraints on the design of experiments to determine speciation during catalysis for this class of catalysts: it is important that the timing of observations be chosen so that the intermediate of interest is present at detectable concentrations. What that timing is depends on how the intermediates are generated (sacrificial oxidant at a particular concentration, light, step in applied potential, the starting oxidation state of the catalyst, *etc.*) and the requirements of the measurements. Simulations such as those described here may be helpful in defining the best time windows.

### 3.5. Coordination and timing, single diad simulations

**Fig. 7:**
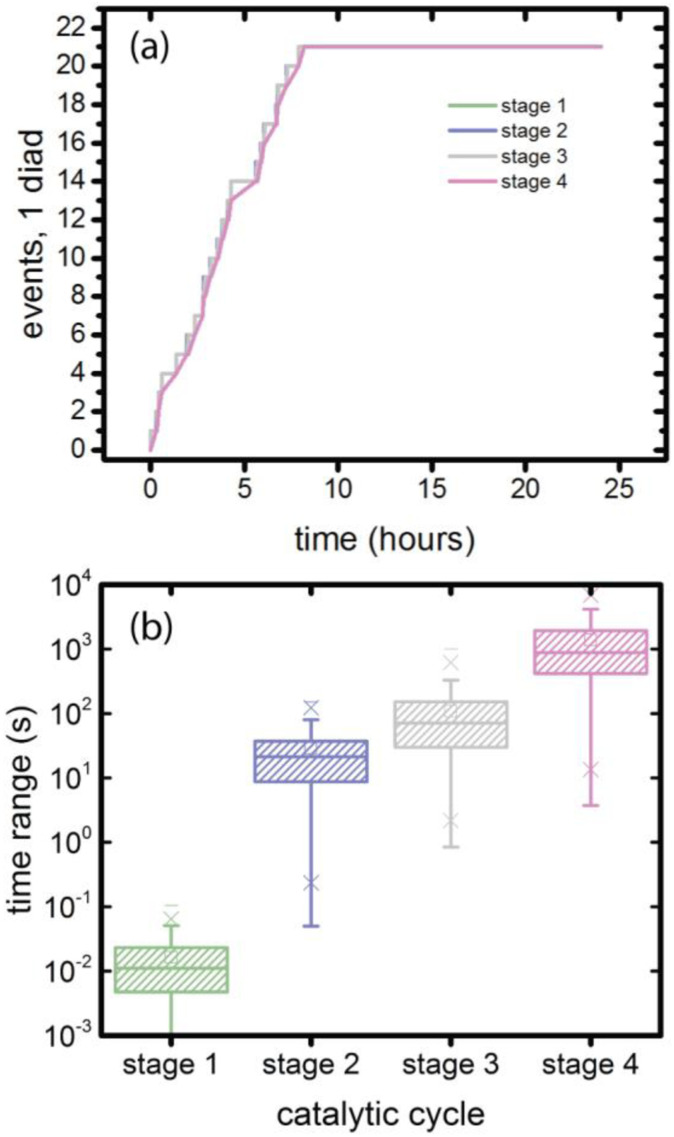
Progression of a single diad through the 4 catalytic stages during 8 hours of illumination followed by 12 hours in the dark. (a) The number of times the diad reaches each stage (typical trace); (b) distribution of times a diad takes to complete each stage (all data).

At the individual diad level, as shown in Fig. 7a, the four catalytic stages are sequential, and that their progress is stochastic in time. This stochasticity can be examined by simulating a large number of 4-stage cycles (397 sets in this case) propagated using 20 different random number strings. The time intervals required to complete a stage are extracted from the simulation results and collected in Fig. 7b and Table 4. The time range for stage 1 reflects only the stochasticity of photoabsorption when the source is diffuse, as is the case for sunlight. The mean value is 0.016 s. Stages 2–4 each involve formation of a non-photoactive intermediate, and the mean time range adds the stochasticity of the chemical reaction rate. Because the mean value for the purely photodriven process is small, the mean values for stages 2–4 are approximately those for the stochasticity of non-photodriven reactions.

**Table 4:** Statistics for characteristic elapsed times for each catalytic stage, 20 distinct single diad simulations, 8 hours of illumination, 16 hours dark, 397 sets

Catalytic stage	Minimum (s)	Maximum (s)	Mean (s)	Standard deviation (s)
1	1.85 × 10^−5^	0.10415	0.016270	0.01573
2	0.04983	150.4268	28.8067	27.4734
3	0.84281	1000.744	109.13253	119.7483
4	3.73195	9464.106	1388.70173	1428.763

## 4 Discussion

The computational study reported here is designed to probe how a dye with characteristic excitation and injection rate coefficients in the range of 10^12^–10^13^ s^−1^, driven by sunlight, and a catalyst attached to it are coordinated to perform photodriven water oxidation through the catalytic cycle. It is clear from the simulations that the formation and accumulation of three diad intermediate states whose reactions cannot be influenced by photoexcitation are central to the timing of catalysis. An ideally-coordinated and efficient photodriven water oxidation reaction will be completely controlled by the dye excitations and charge injections,^23^ much like two perfectly meshed gears: any thermal steps would be instantaneous. Photoexcitations and charge injection under solar flux are sporadic due to its diffuse nature.^12,13^ The only stage in the water oxidation cycle that does not involve a chemical reaction is the first one, where Ru^II^–Ru^II^(OH_2_) is converted to Ru^II^–Ru^III^(OH_2_). Fig. 7b and Table 4 show that the mean time to complete stage 1, *t*_1_, is 0.016 s. If coordination were ideal, this is the expected value that all 4 stages would have, and the mean time *t* for a single diad to complete a cycle in this case is given by 
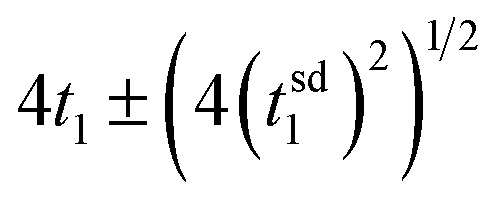
 where *t*_1_ is the characteristic mean time of stage 1 and *t*^sd^_1_ is the standard deviation. For this system, *t* = 0.064 ± 0.031 s. This corresponds to a TOF of about 15 s^−1^, as noted above. Because thermal chemical steps dominate the catalysis, each stage requires significantly more time to be completed, with the slowest (and the one with the largest standard deviation) being the final O_2_ release step. The stochastics of the chemistry dominates the catalysis, increasing the mean time needed to complete a cycle to 1389 ± 1429 s, reducing the TOF to a very small average value. Returning to the meshed gear analogy, in this system the gears' rotation frequencies are unmatched and there are teeth missing. The results of this work point to a potential benefit of designing catalysts so that all intermediates in the cycle can be photo- or charge-activated, removing the stochasticity of chemical reactions near ambient temperature.

That the Ru^II^–Ru^IV^(OO) dissociation step is predicted to be the slowest step is a result of the rate coefficients determined for this catalyst. Buildup of significant quantities of this intermediate in the simulations is consistent with observations of bulk electrolysis of the catalyst, Ru^II^(OH_2_).^25^ Other investigations of molecular water oxidation using this family of catalysts have not unanimously distinguished between stage 3 and stage 4 as rate limiting due to the complex kinetics involved in the experiments.^16–18,36,43,45^ The simulations show that the established kinetics are most consistent with the peroxy species being dominant.

While the present results are obtained using a diad, where redox steps require only direct charge transfer processes, they raise interesting, broader questions about the nature of coordination in photocathodes as well as photoanodes. For example, there are other dye-catalyst configurations for water oxidation reactions involving separately adsorbed dyes and catalysts, where charge transfer is substrate or intramolecularly mediated.^46,47^ In this case an additional timing element would affect the efficiency: charge transfer steps involving transport into and out of defects and surface states in the semiconductor or between the catalyst and a mediator.^48,49^ Catalysis driven by photoexcitations of the substrate only would be a simpler case, where coordination between delocalized excitations and charge transport coupled to catalyst redox steps would be buffered by interfacial traps.^1,3,41^

The simulation results also raise interesting questions about the dyes. What is happening to them when the reaction step in question does not involve them specifically? They will certainly continue to undergo electronic excitations and likely charge injection. How might that energy, which decays to heat, influence the reactivity of the adjacent catalyst? Would charge injection events disrupt the catalytic chemistry? If this occurs reduction of the dye back to its Ru^II^ state must be very fast since very low populations of Ru^III^ species are reported in the most directly related experimental studies.^5^ Although BET is not competitive with catalysis during the photodriven catalytic cycle, it is possible that injection and rapid BET are important processes. BET has been pointed to as the origin of inefficiency in light-driven catalysis. The present work shows that this is unlikely, however its rate could be substantial as a side-process due to the chemical inefficiency of the catalytic stages themselves.

## 5 Conclusions

A detailed stochastic chemical kinetics study of solar-drive water oxidation diad consisting of a Ru dye linked to a semiconductor surface and a Ru catalyst is used to examine how the separate components of the diad work together. The level of coordination of the processes of the two parts of the diad governs their efficiency. Simulations of both ensembles of diads (2 × 10^−10^ moles) and single diads provide insights to average trends as well as the stochasticity of the processes that control how the dye and catalyst interact. The calculations show that the diffuse nature of solar irradiation and its relatively low intensity where the dyes absorb are not rate limiting. They also show that loss processes such as back electron transfer and radiative and non-radiative relaxations are not significant. What they clearly reveal is that because the water oxidation catalysis process involves both thermal and electron-transfer stimulated steps, there is a buildup of those diad intermediates that undergo thermal chemistry. They are formed at several stages of the catalysis, and progression through those stages to steady state requires 500–1000 s. Calculations for single diads provide information on the characteristic timings for the 4 photo-initiated stages of the catalysis. The average time for a diad to complete a purely photodriven step (no reactions with water) is 0.016 s, suggesting that a completely ideal diad with very fast thermal reactions would have a turnover frequency of 15 s^−1^ in sunlight. In reality the calculated turnover frequency is much lower due to the lack of means to accelerate the thermal processes. This study focused on the diad, and can be extended to other dye-catalyst arrangements such as adsorbed mixtures and to catalysts adsorbed on semiconductor supports to characterize how the presence of an indirect charge transfer route influences how well dye and catalyst can coordinate their functions.

## Data availability

The data supporting this study is available within the main text and the associated ESI.†

## Author contributions

Conceptualization: FAH. Investigation (calculations): RM, FAH, TPC. Formal Analysis, Validation, Visualization, Writing: all authors. Funding acquisition: FAH.

## Conflicts of interest

There are no conflicts of interest to declare.

## Supplementary Material

SC-014-D2SC06966K-s001

SC-014-D2SC06966K-s002
