# Supplementary material for: Water oxidation by a dye-catalyst diad in natural sunlight: timing and coordination of excitations and reactions across timescales of picoseconds to hours
Source: Chem Sci. 2023 Jan 23;14(8):1997–2008. doi: 10.1039/d2sc06966k (PMC9945043; doi:10.1039/d2sc06966k)
Supplement: SC-014-D2SC06966K-s001 [file SC-014-D2SC06966K-s001.pdf]

Electronic Supplementary Material (ESI) for Chemical Science.
This journal is © The Royal Society of Chemistry 2023

Electronic Supplementary Information

Water oxidation by a dye-catalyst diad in natural sunlight: timing and coordination of excitations and reactions across timescales of picoseconds to seconds

Ramzi N. Massad,[1,2] Thomas P. Cheshire,[2] Chenqi Fan,[1,2] and Frances A. Houle[2]*

[1] College of Chemistry, University of California, Berkeley, Berkeley, CA 94720, USA.
[2] Chemical Sciences Division, Lawrence Berkeley National Laboratory, Berkeley, CA 94720, USA.

*Corresponding author: fahoule@lbl.gov

Table of Contents:

1. Reaction Scheme……………………………………………………………S2
2. Additional Figure……..……………………………………………………S7
3. References………………………………………………………………..S8

1. Reaction Scheme

The reaction steps listed in Table S1 have the stoichiometries of the transformations of the species, written using the mnemonic dye – catalyst(oxidation state)-oxygen form when the dye is in the ground state, and dye-(dye electronic state) – catalyst(oxidation state)-oxygen form when the dye is in an electronically excited singlet state (X*, B* or Y*) or triplet state (TN*). Each step includes a marker species (in italics) which does not influence the chemistry being simulated, but allows the occurrences of that particular step to be counted. This enables a much deeper analysis of the simulation results.

Table S1. Complete reaction scheme used for the simulations.

Process	Process	Kinetic step	Rate coefficient [a]
1st catalytic cycle	Dye photoexcitation	Dye-cat(2)-OH2 => Dye-TN*-cat(2)-OH2 + *excitation-TN1*	18.3 s^{-1}
	Dye photoexcitation	Dye-cat(2)-OH2 => Dye-Y*-cat(2)-OH2 + *excitation-Y1*	4.28 s^{-1}
	Dye photoexcitation	Dye-cat(2)-OH2 => Dye-B*-cat(2)-OH2 + *excitation-B1*	13.2 s^{-1}
	Dye photoexcitation	Dye-cat(2)-OH2 => Dye-X*-cat(2)-OH2 + *excitation-X1*	22.8 s^{-1}
	Dye photoexcitation	Dye-cat(2)-OH2 => Dye-cat(2)-OH2 + *groundstatebleach-TN1*	18.3 s^{-1}
	Dye photoexcitation	Dye-cat(2)-OH2 => Dye-cat(2)-OH2 + *groundstatebleach-Y1*	4.28 s^{-1}
	Dye photoexcitation	Dye-cat(2)-OH2 => Dye-cat(2)-OH2 + *groundstatebleach-B1*	13.2 s^{-1}
	Dye photoexcitation	Dye-cat(2)-OH2 => Dye-cat(2)-OH2 + *groundstatebleach-X1*	22.8 s^{-1}
	Dye photoexcitation	Dye-TN*-cat(2)-OH2 => Dye-cat(2)-OH2 + *emission-TN1*	18.3 s^{-1}
	Dye photoexcitation	Dye-Y*-cat(2)-OH2 => Dye-cat(2)-OH2 + *emission-Y1*	4.28 s^{-1}
	Dye photoexcitation	Dye-B*-cat(2)-OH2 => Dye-cat(2)-OH2 + *emission-B1*	13.2 s^{-1}
	Dye photoexcitation	Dye-X*-cat(2)-OH2 => Dye-cat(2)-OH2 + *emission-X1*	22.8 s^{-1}
	Dye internal conversion	Dye-Y*-cat(2)-OH2 => Dye-B*-cat(2)-OH2 + *IC_B1*	$2.4x10^{13}$ s^{-1}
	Dye internal conversion	Dye-B*-cat(2)-OH2 => Dye-X*-cat(2)-OH2 + *IC_X1*	$2.4x10^{13}$ s^{-1}
	Dye intersystem crossing	Dye-X*-cat(2)-OH2 => Dye-TN*-cat(2)-OH2 + *ISC1*	4 x 10^{13} s^{-1}
	Dye photoexcitation	Dye-TN*-cat(2)-OH2 => Dye-TN*-cat(2)-OH2 + *esa-TN1*	117 s^{-1}
	Dye photoexcitation	Dye-Y*-cat(2)-OH2 => Dye-Y*-cat(2)-OH2 + *esa-Y1*	117 s^{-1}
	Dye photoexcitation	Dye-B*-cat(2)-OH2 => Dye-B*-cat(2)-OH2 + *esa-B1*	117 s^{-1}

	Dye photoexcitation	Dye-X*-cat(2)-OH2 => Dye-X*-cat(2)-OH2 + *esa-X1*	117 s^{-1}
	Dye radiative relaxation	Dye-TN*-cat(2)-OH2 => Dye-cat(2)-OH2 + *incoherentems-1*	$9.6x10^4$ s^{-1}
	Dye non-radiative relaxation	Dye-TN*-cat(2)-OH2 => Dye-cat(2)-OH2 + *nr-ems-1*	$2.6x10^6$ s^{-1}
	Dye – substrate injection	Dye-TN*-cat(2)-OH2 => Dye+-cat(2)-OH2 + *electron* + *injection-TN1*	$1.0x10^{12}$ s^{-1}
	Dye – substrate injection	Dye-Y*-cat(2)-OH2 => Dye+-cat(2)-OH2 + *electron* + *injection-Y1*	$1.0x10^{12}$ s^{-1}
	Dye – substrate injection	Dye-B*-cat(2)-OH2 => Dye+-cat(2)-OH2 + *electron* + *injection-B1*	$1.0x10^{12}$ s^{-1}
	Dye – substrate injection	Dye-X*-cat(2)-OH2 => Dye+-cat(2)-OH2 + *electron* + *injection-X1*	$1.0x10^{12}$ s^{-1}
	Dye-catalyst electron transfer	Dye+-cat(2)-OH2 => Dye-cat(3)-OH2 + *groundstate1*	6.9 x 10^9 s^{-1} [1]
	Back electron transfer	Dye+-cat(2)-OH2 + electron => Dye-cat(2)-OH2 + *BETox1*	100 s^{-1} [2]
	Back electron transfer	Dye-cat(3)-OH2 + electron => Dye-cat(2)-OH2 + *BETcat1*	100 s^{-1} [2]
2nd catalytic cycle	Dye photoexcitation	Dye-cat(3)-OH2 => Dye-TN*-cat(3)-OH2 + *excitation-TN2*	18.3 s^{-1}
	Dye photoexcitation	Dye-cat(3)-OH2 => Dye-Y*-cat(3)-OH2 + *excitation-Y2*	4.28 s^{-1}
	Dye photoexcitation	Dye-cat(3)-OH2 => Dye-B*-cat(3)-OH2 + *excitation-B2*	13.2 s^{-1}
	Dye photoexcitation	Dye-cat(3)-OH2 => Dye-X*-cat(3)-OH2 + *excitation-X2*	22.8 s^{-1}
	Dye photoexcitation	Dye-cat(3)-OH2 => Dye-cat(3)-OH2 + *groundstatebleach-TN2*	18.3 s^{-1}
	Dye photoexcitation	Dye-cat(3)-OH2 => Dye-cat(3)-OH2 + *groundstatebleach-Y2*	4.28 s^{-1}
	Dye photoexcitation	Dye-cat(3)-OH2 => Dye-cat(3)-OH2 + *groundstatebleach-B2*	13.2 s^{-1}
	Dye photoexcitation	Dye-cat(3)-OH2 => Dye-cat(3)-OH2 + *groundstatebleach-X2*	22.8 s^{-1}
	Dye photoexcitation	Dye-TN*-cat(3)-OH2 => Dye-cat(3)-OH2 + *emission-TN2*	18.3 s^{-1}
	Dye photoexcitation	Dye-Y*-cat(3)-OH2 => Dye-cat(3)-OH2 + *emission-Y2*	4.28 s^{-1}
	Dye photoexcitation	Dye-B*-cat(3)-OH2 => Dye-cat(3)-OH2 + *emission-B2*	13.2 s^{-1}
	Dye photoexcitation	Dye-X*-cat(3)-OH2 => Dye-cat(3)-OH2 + *emission-X2*	22.8 s^{-1}
	Dye internal conversion	Dye-Y*-cat(3)-OH2 => Dye-B*-cat(3)-OH2 + *IC_B2*	$2.4x10^{13}$ s^{-1}
	Dye internal conversion	Dye-B*-cat(3)-OH2 => Dye-X*-cat(3)-OH2 + *IC_X2*	$2.4x10^{13}$ s^{-1}
	Dye intersystem crossing	Dye-X*-cat(3)-OH2 => Dye-TN*-cat(3)-OH2 + *ISC2*	4 x 10^{13} s^{-1}
	Dye photoexcitation	Dye-TN*-cat(3)-OH2 => Dye-TN*-cat(3)-OH2 + *esa-TN2*	117 s^{-1}

	Dye photoexcitation	Dye-Y*-cat(3)-OH2 => Dye-Y*-cat(3)-OH2 + *esa-Y2*	117 s^{-1}
	Dye photoexcitation	Dye-B*-cat(3)-OH2 => Dye-B*-cat(3)-OH2 + *esa-B2*	117 s^{-1}
	Dye photoexcitation	Dye-X*-cat(3)-OH2 => Dye-X*-cat(3)-OH2 + *esa-X2*	117 s^{-1}
	Dye radiative relaxation	Dye-TN*-cat(3)-OH2 => Dye-cat(3)-OH2 + *incoherentems-2*	$9.6x10^4$ s^{-1}
	Dye non-radiative relaxation	Dye-TN*-cat(3)-OH2 => Dye-cat(3)-OH2 + *nr-ems-2*	$2.6x10^6$ s^{-1}
	Dye – substrate injection	Dye-TN*-cat(3)-OH2 => Dye+-cat(3)-OH2 + *electron* + *injection-TN2*	$1.0x10^{12}$ s^{-1}
	Dye – substrate injection	Dye-Y*-cat(3)-OH2 => Dye+-cat(3)-OH2 + *electron* + *injection-Y2*	$1.0x10^{12}$ s^{-1}
	Dye – substrate injection	Dye-B*-cat(3)-OH2 => Dye+-cat(3)-OH2 + *electron* + *injection-B2*	$1.0x10^{12}$ s^{-1}
	Dye – substrate injection	Dye-X*-cat(3)-OH2 => Dye+-cat(3)-OH2 + *electron* + *injection-X2*	$1.0x10^{12}$ s^{-1}
	1 electron 2 proton transfer	Dye+-cat(3)-OH2 => Dye-cat(4)--O + 2 H+ + *protonrelease*	0.036 s^{-1} [3]
	Back electron transfer	Dye+-cat(2)-OH2 + electron => Dye-cat(2)-OH2 + *BETox1*	100 s^{-1} [2]
3rd catalytic cycle	Dye photoexcitation	Dye-cat(4)--O => Dye-TN*- cat(4)--O + *excitation-TN3*	18.3 s^{-1}
	Dye photoexcitation	Dye-cat(4)--O => Dye-Y*- cat(4)--O + *excitation-Y3*	4.28 s^{-1}
	Dye photoexcitation	Dye-cat(4)--O => Dye-B*- cat(4)--O + *excitation-B3*	13.2 s^{-1}
	Dye photoexcitation	Dye-cat(4)--O => Dye-X*- cat(4)--O + *excitation-X3*	22.8 s^{-1}
	Dye photoexcitation	Dye-cat(4)--O => Dye-cat(4)--O + *groundstatebleach-TN3*	18.3 s^{-1}
	Dye photoexcitation	Dye-cat(4)--O => Dye-cat(4)--O + *groundstatebleach-Y3*	4.28 s^{-1}
	Dye photoexcitation	Dye-cat(4)--O => Dye-cat(4)--O + *groundstatebleach-B3*	13.2 s^{-1}
	Dye photoexcitation	Dye-cat(4)--O => Dye-cat(4)--O + *groundstatebleach-X3*	22.8 s^{-1}
	Dye photoexcitation	Dye-TN*- cat(4)--O => Dye-cat(4)--O + *emission-TN3*	18.3 s^{-1}
	Dye photoexcitation	Dye-Y*- cat(4)--O => Dye-cat(4)--O + *emission-Y3*	4.28 s^{-1}
	Dye photoexcitation	Dye-B*- cat(4)--O => Dye-cat(4)--O + *emission-B3*	13.2 s^{-1}
	Dye photoexcitation	Dye-X*- cat(4)--O => Dye-cat(4)--O + *emission-X3*	22.8 s^{-1}
	Dye internal conversion	Dye-Y*- cat(4)--O => Dye-B*- cat(4)--O + *IC_B3*	$2.4x10^{13}$ s^{-1}
	Dye internal conversion	Dye-B*- cat(4)--O => Dye-X*- cat(4)--O + *IC_X3*	$2.4x10^{13}$ s^{-1}
	Dye intersystem crossing	Dye-X*- cat(4)--O => Dye-TN*- cat(4)--O + *ISC3*	4 x 10^{13} s^{-1}

	Dye photoexcitation	Dye-TN*- cat(4)--O => Dye-TN*- cat(4)--O + *esa-TN3*	117 s^{-1}
	Dye photoexcitation	Dye-Y*- cat(4)--O => Dye-Y*- cat(4)--O + *esa-Y3*	117 s^{-1}
	Dye photoexcitation	Dye-B*- cat(4)--O => Dye-B*- cat(4)--O + *esa-B3*	117 s^{-1}
	Dye photoexcitation	Dye-X*- cat(4)--O => Dye-X*- cat(4)--O + *esa-X3*	117 s^{-1}
	Dye radiative relaxation	Dye-TN*- cat(4)--O => Dye-cat(4)--O + *incoherentems-3*	9.6×10^{4} s^{-1}
	Dye non-radiative relaxation	Dye-TN*- cat(4)--O => Dye-cat(4)--O + *nr-ems-3*	2.6×10^{6} s^{-1}
	Dye – substrate injection	Dye-TN*- cat(4)--O => Dye+-cat(4)--O + electron + *injection-TN3*	1.0×10^{12} s^{-1}
	Dye – substrate injection	Dye-Y*- cat(4)--O => Dye+-cat(4)--O + electron + *injection-Y3*	1.0×10^{12} s^{-1}
	Dye – substrate injection	Dye-B*- cat(4)--O => Dye+-cat(4)--O + electron + *injection-B3*	1.0×10^{12} s^{-1}
	Dye – substrate injection	Dye-X*- cat(4)--O => Dye+-cat(4)--O + electron + *injection-X3*	1.0×10^{12} s^{-1}
	Dye-catalyst electron transfer	Dye+-cat(4)--O => Dye-cat(5)--O + *groundstate3*	6.9×10^{9} s^{-1} [1]
	O-atom proton transfer	Dye-cat(5)--O => Dye-cat(3)-OOH + H+ + *OOformation*	0.0096 s^{-1} [4]
	Back electron transfer	Dye+-cat(4)--O + electron => Dye-cat(4)--O + *BETox3*	100 s^{-1} [2]
	Back electron transfer	Dye-cat(5)--O + electron => Dye-cat(4)--O + *BETcat3*	100 s^{-1} [2]
4th catalytic cycle	Dye photoexcitation	Dye-cat(3)-OOH => Dye-TN*-cat(3)-OOH + *excitation-TN4*	18.3 s^{-1}
	Dye photoexcitation	Dye-cat(3)-OOH => Dye-Y*-cat(3)-OOH + *excitation-Y4*	4.28 s^{-1}
	Dye photoexcitation	Dye-cat(3)-OOH => Dye-B*-cat(3)-OOH + *excitation-B4*	13.2 s^{-1}
	Dye photoexcitation	Dye-cat(3)-OOH => Dye-X*-cat(3)-OOH + *excitation-X4*	22.8 s^{-1}
	Dye photoexcitation	Dye-cat(3)-OOH => Dye-cat(3)-OOH + *groundstatebleach-TN4*	18.3 s^{-1}
	Dye photoexcitation	Dye-cat(3)-OOH => Dye-cat(3)-OOH + *groundstatebleach-Y4*	4.28 s^{-1}
	Dye photoexcitation	Dye-cat(3)-OOH => Dye-cat(3)-OOH + *groundstatebleach-B4*	13.2 s^{-1}
	Dye photoexcitation	Dye-cat(3)-OOH => Dye-cat(3)-OOH + *groundstatebleach-X4*	22.8 s^{-1}
	Dye photoexcitation	Dye-TN*-cat(3)-OOH => Dye-cat(3)-OOH + *emission-TN4*	18.3 s^{-1}
	Dye photoexcitation	Dye-Y*-cat(3)-OOH => Dye-cat(3)-OOH + *emission-Y4*	4.28 s^{-1}
	Dye photoexcitation	Dye-B*-cat(3)-OOH => Dye-cat(3)-OOH + *emission-B4*	13.2 s^{-1}
	Dye photoexcitation	Dye-X*-cat(3)-OOH => Dye-cat(3)-OOH + *emission-X4*	22.8 s^{-1}

	Dye internal conversion	Dye-Y*-cat(3)-OOH => Dye-B*-cat(3)-OOH + *IC_B4*	$2.4x10^{13}$ s^{-1}
	Dye internal conversion	Dye-B*-cat(3)-OOH => Dye-X*-cat(3)-OOH + *IC_X4*	$2.4x10^{13}$ s^{-1}
	Dye intersystem crossing	Dye-X*-cat(3)-OOH => Dye-TN*-cat(3)-OOH + *ISC4*	4×10^{13} s^{-1}
	Dye photoexcitation	Dye-TN*-cat(3)-OOH => Dye-TN*-cat(3)-OOH + *esa-TN4*	117 s^{-1}
	Dye photoexcitation	Dye-Y*-cat(3)-OOH => Dye-Y*-cat(3)-OOH + *esa-Y4*	117 s^{-1}
	Dye photoexcitation	Dye-B*-cat(3)-OOH => Dye-B*-cat(3)-OOH + *esa-B4*	117 s^{-1}
	Dye photoexcitation	Dye-X*-cat(3)-OOH => Dye-X*-cat(3)-OOH + *esa-X4*	117 s^{-1}
	Dye radiative relaxation	Dye-TN*-cat(3)-OOH => Dye-cat(3)-OOH + *incoherentems-4*	$9.6x10^{4}$ s^{-1}
	Dye non-radiative relaxation	Dye-TN*-cat(3)-OOH => Dye-cat(3)-OOH + *nr-ems-4*	$2.6x10^{6}$ s^{-1}
	Dye – substrate injection	Dye-TN*-cat(3)-OOH => Dye+-cat(3)-OOH + electron + *injection-TN4*	$1.0x10^{12}$ s^{-1}
	Dye – substrate injection	Dye-Y*-cat(3)-OOH => Dye+-cat(3)-OOH + electron + *injection-Y4*	$1.0x10^{12}$ s^{-1}
	Dye – substrate injection	Dye-B*-cat(3)-OOH => Dye+-cat(3)-OOH + electron + *injection-B4*	$1.0x10^{12}$ s^{-1}
	Dye – substrate injection	Dye-X*-cat(3)-OOH => Dye+-cat(3)-OOH + electron + *injection-X4*	$1.0x10^{12}$ s^{-1}
	Dye-catalyst electron transfer	Dye+-cat(3)-OOH => Dye-cat(4)-OO + H+ + *groundstate4*	6.9×10^{9} s^{-1} [1]
	Oxygen release	Dye-cat(4)-OO => Dye-cat(2)-OH2 + O2 + *watersplitting*	7.5×10^{-4} s^{-1} [4]
	Back electron transfer	Dye+-cat(3)-OOH + electron => Dye-cat(3)-OOH + *BETox4*	100 s^{-1} [2]
	Back electron transfer	Dye-cat(4)-OO + electron => Dye-cat(3)-OOH + *BETcat4*	100 s^{-1} [2]

[a] Rate coefficients for dye excitations calculated assuming dye-catalyst complexes are adsorbed inside a nanoparticulate TiO_2 matrix, including optical scattering. All optical transitions are pseudo-first order in absorbed photon flux. All dye photophysical and charge injection rate coefficients are from Ref [5].

2. Additional figures

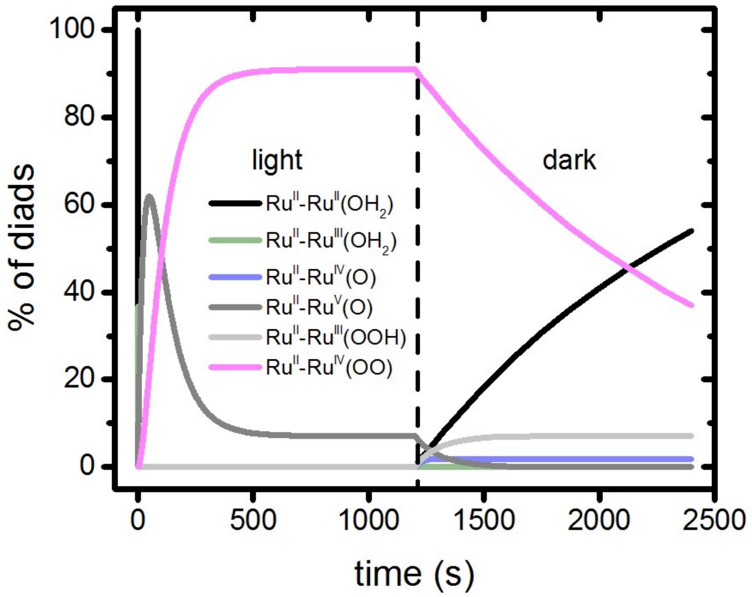

Figure S1. Linear y axis version of Figure 2c, main manuscript. Predictions by simulations in a 1200s light – 1200s dark sequence, diads present with dye in the Ru^{II} state. The colors correspond to the species present in each catalytic stage, Figure 1, main manuscript.

3. References

1. L. Wang, D. L. Ashford, D. W. Thompson, T. J. Meyer and J. M. Papanikolas, *J Phys Chem C*, 2013, **117**, 24250-24258.
2. P. T. Xu, C. L. Gray, L. Q. Xiao and T. E. Mallouk, *J Am Chem Soc*, 2018, **140**, 11647-11654.
3. K. Hu, R. N. Sampaio, S. L. Marquard, M. K. Brennaman, Y. Tamaki, T. J. Meyer and G. J. Meyer, *Inorg Chem*, 2018, **57**, 486-494.
4. J. J. Concepcion, M. K. Tsai, J. T. Muckerman and T. J. Meyer, *J Am Chem Soc*, 2010, **132**, 1545-1557.
5. T. P. Cheshire, J. Boodry, E. A. Kober, M. K. Brennaman, P. G. Giokas, D. F. Zigler, A. M. Moran, J. M. Papanikolas, G. J. Meyer, T. J. Meyer and F. A. Houle, *J Chem Phys*, 2022 DOI: 10.1063/5.0127852.
